# Malaria risk in Nigeria: Bayesian geostatistical modelling of 2010 malaria indicator survey data

**DOI:** 10.1186/s12936-015-0683-6

**Published:** 2015-04-14

**Authors:** Abbas B Adigun, Efron N Gajere, Olusola Oresanya, Penelope Vounatsou

**Affiliations:** Department of Public Health and Epidemiology, Swiss Tropical and Public Health Institute, P.O. Box 4002 Basel, Switzerland; University of Basel, Petersplatz 1, 4051 Basel, Switzerland; National Malaria Control Programme, Abuja, Nigeria; National Centre for Remote Sensing, Jos, Nigeria

**Keywords:** Bayesian variable selection, Bayesian inference, Markov Chain Monte Carlo, Parasitaemia, Malaria risk, Control intervention

## Abstract

**Background:**

In 2010, the National Malaria Control Programme with the support of Roll Back Malaria partners implemented a nationally representative Malaria Indicator Survey (MIS), which assembled malaria burden and control intervention related data. The MIS data were analysed to produce a contemporary smooth map of malaria risk and evaluate the control interventions effects on parasitaemia risk after controlling for environmental/climatic, demographic and socioeconomic characteristics.

**Methods:**

A Bayesian geostatistical logistic regression model was fitted on the observed parasitological prevalence data. Important environmental/climatic risk factors of parasitaemia were identified by applying Bayesian variable selection within geostatistical model. The best model was employed to predict the disease risk over a grid of 4 km^2^ resolution. Validation was carried out to assess model predictive performance. Various measures of control intervention coverage were derived to estimate the effects of interventions on parasitaemia risk after adjusting for environmental, socioeconomic and demographic factors.

**Results:**

Normalized difference vegetation index and rainfall were identified as important environmental/climatic predictors of malaria risk. The population adjusted risk estimates ranges from 6.46% in Lagos state to 43.33% in Borno. Interventions appear to not have important effect on malaria risk. The odds of parasitaemia appears to be on downward trend with improved socioeconomic status and living in rural areas increases the odds of testing positive to malaria parasites. Older children also have elevated risk of malaria infection.

**Conclusions:**

The produced maps and estimates of parasitaemic children give an important synoptic view of current parasite prevalence in the country. Control activities will find it a useful tool in identifying priority areas for intervention.

**Electronic supplementary material:**

The online version of this article (doi:10.1186/s12936-015-0683-6) contains supplementary material, which is available to authorized users.

## Background

Malaria represents a substantial public health challenge in Nigeria and is a major cause of morbidity and mortality. The country accounts for up to 25% of malaria burden in sub-Saharan Africa, which is globally the highest burden region for malaria [1]. In terms of morbidity, around 110 million of clinically diagnosed cases, 30 percent of health care facilities admission and 60 percent of outpatient visits are attributed to the disease each year [2]. Malaria is also responsible for 300,000 childhood deaths and 11% maternal deaths annually [1,3,4].

Control of malaria is hinged on key global strategies, which include prompt and effective case management, intermittent preventive treatment (IPT) of malaria in pregnancy and integrated vector management (IVM) comprising the use of insecticide-treated nets (ITN), indoor residual spraying (IRS), and environmental management (EM).

The National Malaria Control Programme (NMCP) with the support of Roll Back Malaria (RBM) partners is keying into these strategies which form the basis of its National Malaria Control Strategic plan (2009-2013) [1]. Long-lasting impregnated net (LLIN) possession was scaled up by mass distribution of more than 24 million LLIN in 14 states of the country as of August 2010 through a campaign supported by the partners [4]. Prior to this campaign, more than 600,000 LLINs have been distributed in Cross River State between late 2008 and early 2009 to children under the age of five by the United State Agency for International Development (USAID) and the Canadian Red Cross [4]. These efforts contributed to about 42 percent of households having at least one ITN [1]. Between 2008 and 2010, 70 million rapid diagnostic tests (RDTs) were distributed to all heath facilities in the country to allow for free diagnosis of all suspected malaria cases [2]. In 2008, 5% of these cases were screened with RDTs [2]. Pregnant women receiving preventive therapy during their routine antenatal care reached 13 percent in 2010, which may reflect low turnout for antenatal visit and at the same time health care-seeking behaviour. At the end of the same year IRS coverage was two percent in the entire country [2].

Effective malaria control strategies call for reliable and comprehensive maps of the spatial distribution of the disease risk and estimates of infected people. These are important tools in guiding efficient resource allocation for planning and implementation of intervention programmes and evaluation of their impact [5-9]. Various maps depicting the geographical distribution of malaria risk in Nigeria are presently available at regional, continental, and global scale. The earlier map of malaria risk in Nigeria was a climatic suitability map estimated by the mapping malaria risk in Africa (MARA) project [10]. This effort was followed up by empirical mapping using historical survey data from the MARA database to produce a regional map of West Africa [5]. Different Bayesian geostatistical modelling approaches were employed to these historical data attempting to improve the model-based prediction of malaria risk. Sequel to this the Malaria Atlas Project (MAP) in 2007 and 2010 generated a geostatistical model-based global malaria risk map from historical survey data [11,12]. More recently, geostatistical model-based spatio-temporal malaria endemicity maps of Africa were obtained through analysis of data assembled from parasite prevalence surveys adjusting for environmental factors effect [13]. Analyses that are based on historical survey data suffer from methodological issues due to data heterogeneity that may contribute to less accurate estimates [6-9] and do not reflect the current situation of the disease in the country.

In 2010, Nigeria conducted the first nationally representative MIS which assembled information on malaria-related burden and the coverage of key interventions among children below the age of five. The survey was implemented by the National Population Commission (NPC) and NMCP with the technical assistance of ICF International and other RBM partners. In this study, the MIS data were analysed in order to identify environmental/climatic, demographic, and socioeconomic and control intervention factors associated with malaria risk and produce a contemporary risk map of malaria among children under the age of five. Bayesian geostatistical models fitted via Markov Chain Monte Carlo (MCMC) simulation were employed for parameter estimation and predictions. Gibbs variable selection incorporating spatial dependency was used in identifying the most parsimonious model.

## Methods

### Study area

Nigeria, the most populous country in Africa, is in the west sub region of Africa with a total land mass of 923, 768 square kilometres. The recent census estimates the country population at 140,431,790 people, 32.8% of which are urban settlers [14]. The country has tropical climate with two seasons (wet and dry season) which are associated with the movement of two dominant winds: the rain bearing south westerly winds, and the cold, dry and dusty north easterly wind generally referred to as the Harmattan. The wet season occurs from April to September, and the dry season from October to March. The annual rainfall ranges between 550mm in some part of the north mainly in the fringes of Sahara desert to 4,000 mm in the coastal region around Niger delta area in the south. The temperature in Nigeria oscillates between 25°C and 40°C.The vegetation that derives from these climatic differences consists of mangrove swamp forest in the Niger Delta to Sahel Savannah in the north. The geographic location of Nigeria makes suitable climate for malaria transmission throughout the country and it is all year round in most part of the country. The most prevalent malaria parasite species is *Plasmodium falciparum* (>95%) and it is responsible for most forms of the severe disease [1,2]. The other types found are *Plasmodium malariae* and *Plasmodium ovale* [2]. Malaria transmission intensity, duration and seasonality vary among the country’s five ecological strata (mangrove swamps, rain forest, guinea savannah, Sudan savannah and Sahel savannah) that extend from south to north [1]. Considering population density and distribution of risk areas, an estimated 3%, 67% and 30% live in very low to low, moderate, and high to very high transmission intensities area, respectively [2]. Also the duration of transmission season increases from north to south, from approximately three months in the north area bordering Chad to perennial in the most southern part [2].

### MIS data

**Figure 1:**
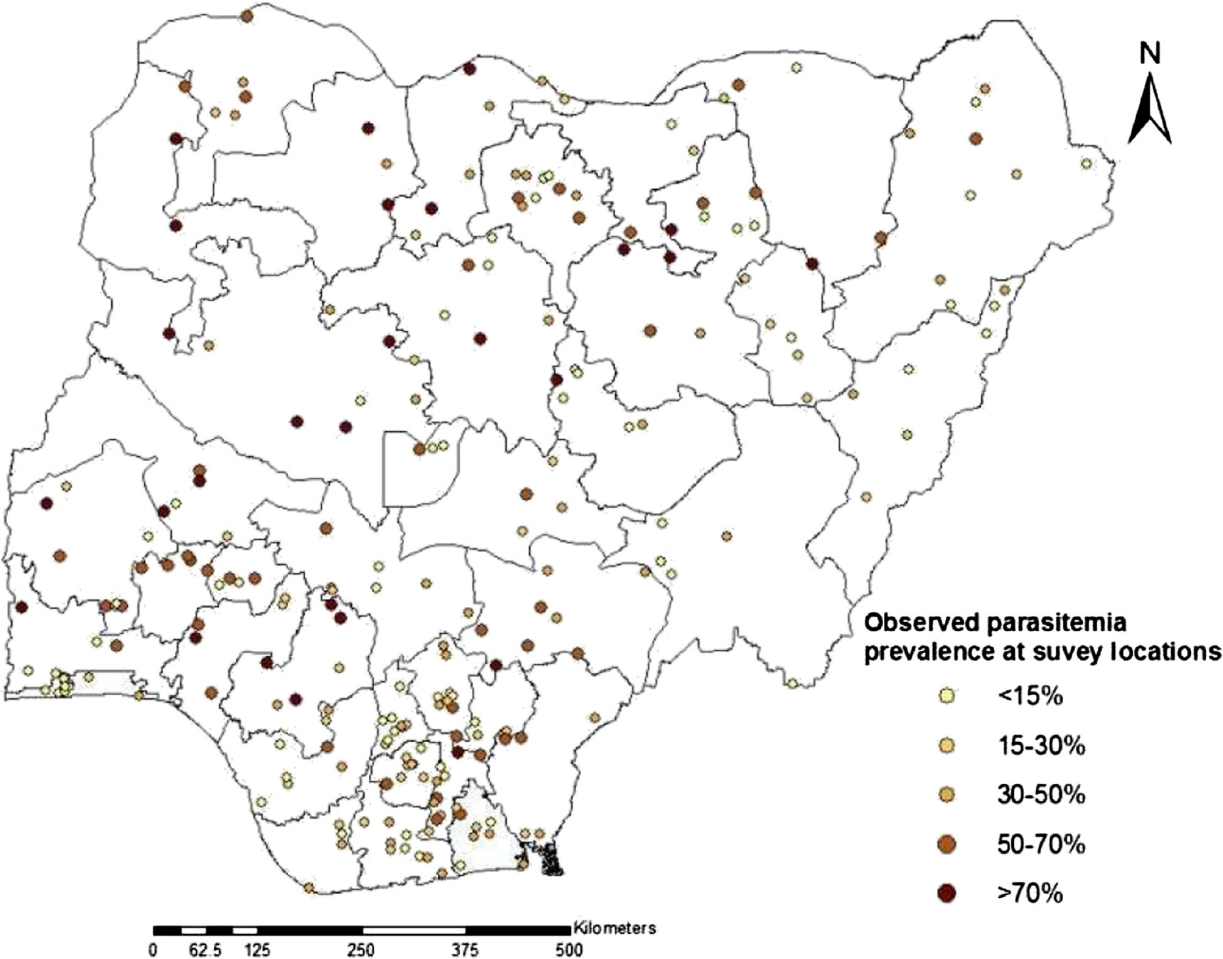
Malaria prevalence observed among children less than 5 years at 239 locations of NMIS in 2010.

The data were collected using the standard malaria indicator questionnaires developed by the RBM and the demographic health surveillance programme. The dataset consists of malariometric information, demographic characteristics and socio-economic status on a nationally representative sample of around 6,000 households from about 240 clusters of which 83 are in the urban areas. These clusters were derived from a stratified two-stage cluster design. Detail description of the sampling strategies is well-documented in the final report of NMIS 2010 [1]. Blood samples were only taken from 239 clusters due to security challenges in one of the clusters in the north [1]. Prevalence from two diagnostic methods (RDT and microscopy) were recorded in the data, but the statistical analysis in this work is based on the blood slide microscopy readings which is believed to be the gold standard of malaria diagnosis [15]. The geographical representation of the clusters involved and observed prevalence in the NMIS is displayed in Figure 1.

### Environmental/climatic data

Environmental and climatic predictors were obtained from satellite sources. The acquired factors used in this analysis are Land Surface Temperature (LST), Normalized Difference Vegetation Index (NDVI), altitude, rainfall and distance to permanent water bodies. Weekly and biweekly values of LST and NDVI, respectively, covering the period from October 2009 to October 2010 were extracted from the Moderate Resolution Imaging Spectroradiometer (MODIS) database [16]. Decadal rainfall data for the same period was downloaded from the Africa Data Dissemination Service database [17]. Annual averages at each location (observed or predicted) were derived for the above predictors. Data on permanent water-bodies was obtained from the HealthMapper database of the World Health Organization (WHO). The minimum distance between the centroid of each cluster to the nearest body of water was calculated in ArcGIS version 9.3 (ESRI; Redlands, CA, USA). The Urban-rural extent grid data was acquired from the Global Rural Urban Mapping Project (GRUMP) database. Details about the sources, spatial and temporal resolution of these data is shown in Table 1. The coordinates of the clusters in the MIS was used to link malaria data with these datasets.

**Table 1:** **Sources, spatial and temporal resolution of model predictors and population data**

**Data**	**Source**	**Period**	**Spatial resolution**
**Land surface temperature (LST)**	MODerate resolution Imaging Spectroradiometer	2009-2010	1 × 1 km^2^
for day and night	(MODIS) http://reverb.echo.nasa.gov/reverb		
**Normalized difference vegetation index**	MODerate resolution Imaging Spectroradiometer	2009-2010	0.25 × 0.25 km^2^
**(NDVI)**	(MODIS) http://reverb.echo.nasa.gov/reverb		
**Rainfall**	Africa Data Disseminating Services	2009-2010	8 × 8 km^2^
	http://earlywarning.usgs.gov/fews/		
**Digital elevation model (Altitude)**	Shuttle Radar Topographic Mission(SRTM)	2000	1 × 1 km^2^
	http://glcfapp.glcf.umd.edu/data/srtm/		
**Urban rural extent**	Global Rural and Urban Mapping Project	na	1 × 1 km^2^
	http://sedac.ciesin.columbia.edu/data/set/grump-v1-population/data-download		
**Permanent water bodies**	Health mapper	na	1 × 1 km^2^
**Human population density grid**	http://www.worldpop.org.uk/data/	2010	100 ×100 m^2^

### Intervention data

Data on measures for preventing malaria, including the possession and use of ITN /LLIN and implementation of IRS were collected in the NMIS. These data were used to generate the following indicators of intervention coverage as recommended by Roll Back Malaria-Measurement and Evaluation Reference Group (RBM-MERG) [18,19]: (i) the proportion with access to ITN in the household, (ii) proportion in every household that slept under an ITN during the previous night to the survey, (iii) proportion of children under 5 who slept under an ITN during the night preceding survey.

### Socioeconomic data

Information on socioeconomic status (SES) was measured by a wealth index, which was present in the NMIS. It was derived through Principal Component Analysis as a weighted sum of household assets. SES was included in the analysis as a categorical covariate with categories corresponding to the quintiles.

### Population data

Population density grid data for the year 2010 was extracted from Worldpop [20]. Population structure for the same year was obtained from international database of United State census bureau [21] to calculate the number of children under five years.

### Bayesian geostatistical modelling

Bayesian geostatistical logistic regression models were applied to identify important predictors of malaria parasite risk, produce a contemporary malaria risk map, and obtain estimates of number of children less than five years old infected with malaria parasites. Variable selection was carried out during the geostatistical model fit. All possible combinations of covariates resulting in 65536 models were fitted to obtain a parsimonious model.

Prediction was carried out using Bayesian kriging [22] based on the model with the best predictive ability. Model validation was performed on the first two models with the highest probability of having generated the data among those considered. In particular, the models were fitted on a random sample of 85% of the locations and used the remaining locations to compare model-based predictions with observed prevalence by calculating the Mean Absolute Error (MAE). A regular grid of 231,865 pixels at 4 km^2^ spatial resolution covering the whole country was generated to predict the parasitaemia risk at unsampled locations and produce a high-resolution risk map. Population data on the number of children under five years of age was combined with spatially explicitly predicted parasitaemia risk to estimate the number of infected children. The analysis was carried out in WinBUG1.4 (Imperial College and Medical Research Council London, United Kingdom). Bayesian kriging was implemented in FORTRAN 95 (Compaq Visual FORTRAN Professional 6.6.0) using standard numerical libraries (NAG, The Numerical Algorithm Group Ltd). Details on Bayesian model selection, model fit and validation are provided in Additional file 1.

## Results

The study included 5,043 children under five years old with complete parasitological and malaria intervention data collected over 239 geo-located clusters. The overall prevalence using thick blood smear results was 38%. On average, one ITN is available for every four children or for every five individuals in the household and only 26% of the children less than age of five slept under an ITN during the night preceding the survey. The IRS coverage is 1.02% in the entire country.

Table 2 shows that the highest model posterior probability was 0.45, that is 45% of the fitted models included rainfall and NDVI in linear forms (Model 1), followed by the one including the above covariates in addition to LST and altitude (posterior probability 0.08) . Model validation results depicted in Table 2 indicate that both models have similar MAE estimates in terms of predictive ability, confirming that the LST and altitude did not improve predictions, therefore, inferences were based on Model 1.

**Table 2:** **Model predictive performance in terms of Mean Absolute Error (MAE) based on climatic/environmental factors**

**Model**	**Posterior probability**	**Mean absolute error**
Rainfall and NDVI	45%	0.005
Rainfall, NDVI, LSTN and Altitude	8%	0.005

Posterior estimates of the parameters of (Model 1) as well as the model which includes environmental/climatic, socio-economic, demographic, and intervention covariates (Model 2) are given in Additional file 2. Higher vegetation index, is associated with high parasitaemia while increased rainfall reduces malaria risk. A monotone decrease of malaria risk was observed with better socio-economic status which becomes important in the stratum of least poor with an odds ratio of 0.51(95%BCI: 0.35 – 0.75). Older children have elevated odds of being infected. Moreover, living in rural areas puts children at higher risk. Furthermore variation in intervention coverage appears not to be associated with parasiteamia risk. The estimates of the range parameter shows that spatial correlation is present within an ~3.0 km (95% BCI:1.50 km - 45.00 km) distance which implies that malaria risk at a given location is affected by risk in neighbouring areas up to a distance of approximately 3.0 km.

**Figure 2:**
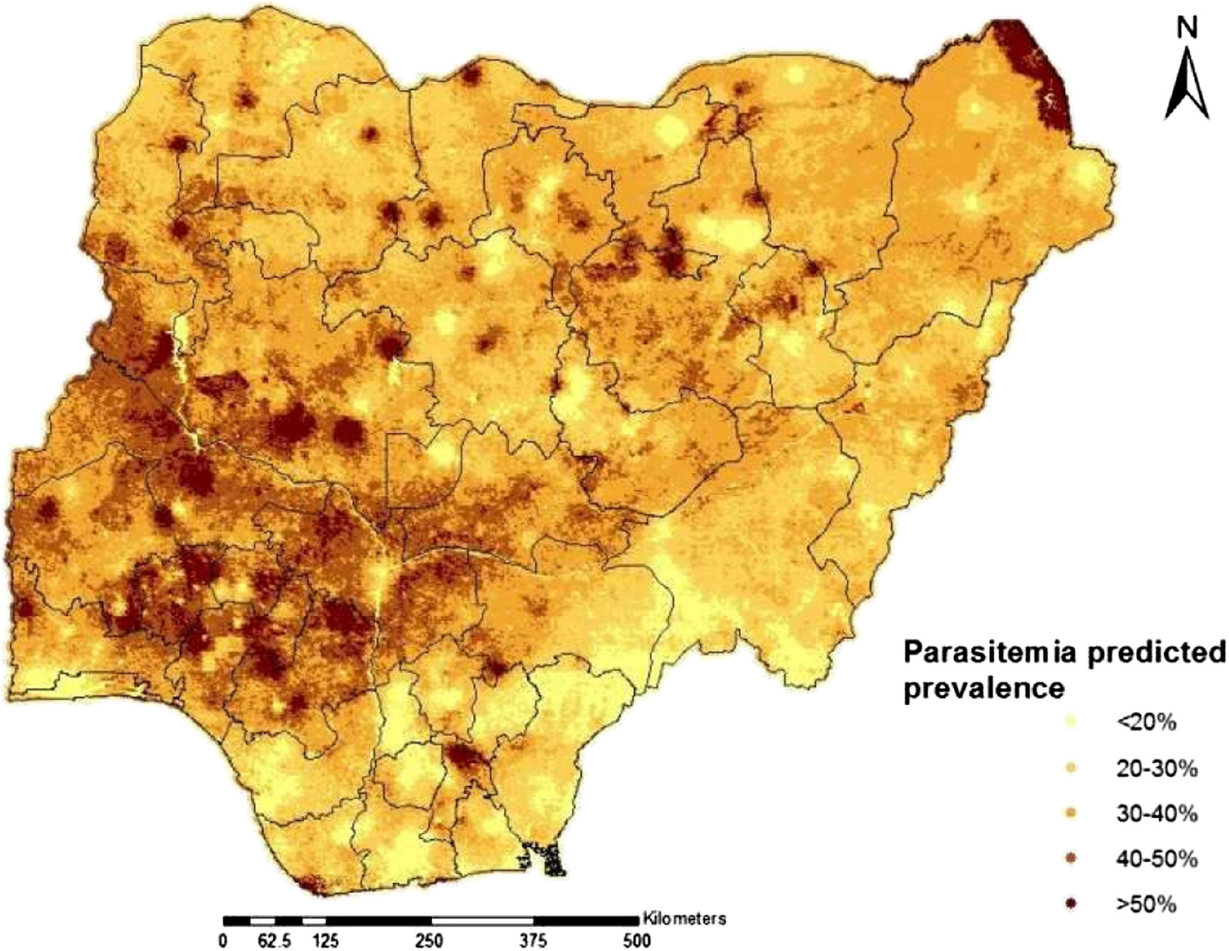
Predicted risk map of parasite among children under 5 year in Nigeria: Estimates are based on Model 1 and indicate median posterior distributions over a grid of 231865 pixels.

**Figure 3:**
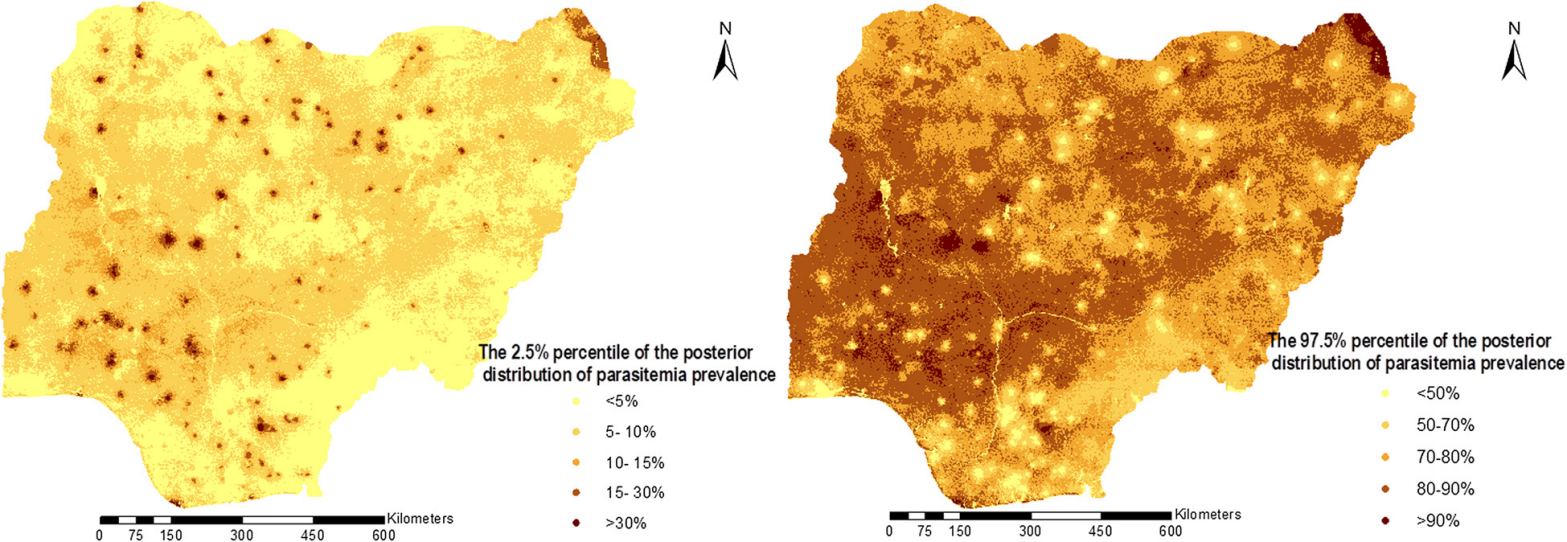
The 2.5 %( left) and 97.5 %( right) percentiles of the predicted posterior distribution of malaria prevalence estimated from Model 1.

Results of a sensitivity analysis showed that the estimates of the spatial parameters were not sensitive to the choice of the priors. The predicted parasitaemia risk map is depicted in Figure 2. The maps of the lower 2.5% and upper 97.5% credible intervals of the posterior distribution are also displayed in Figure 3.

The distribution of predicted parasitaemia risk varies in Nigeria between 0.4% and 91%. Parasitaemia prevalence is relatively low in the southern-most and the south-east region of the country particularly in Anambra state where the risk of testing positive to parasitaemia is below 20%. It is only in Abia and Edo states within those regions that the parasitaemia risk respectively went above 30% and slightly above 40%. The south-west, with exception of Lagos state shows relatively higher risk, however the highest prevalence in the country (~48%) was predicted for Osun state within this region. The situation in the central north is similar to the south-west with Kwara and Benue states having the highest (42.4%) and lowest (29.7%) risk in this region, respectively. Most of the state in the north-east and north-west exhibit similar patterns with the exception of Yobe state with parasitaemia risk of approximately 40%.

Estimates of the population adjusted prevalence and number of parasitemic children under 5 year aggregated at the state level are given in Additional file 3. Overall 7,104,079 children were estimated to be infected with malaria parasites distributed across regions as follows: 16.5% north-central, 15.6% north-east, 26.7% north-west, 9.80% south-east, 13.1% southern-most, and 18.4% south-west.

## Discussion

This work present a geostatistical analysis of the NMIS data to identify important predictors of malaria parasite risk, produce a contemporary malaria risk map, and obtain estimates of number of children less than 5 years old infected with malaria parasites. It is noteworthy that this study generated the first spatially referenced parasitaemia risk estimates and maps in Nigeria from contemporary, geographically-representative data collected in a standardized way across the country. Previous mapping efforts embedded Nigeria within regional, continental and global scale [5,12,13,23] maps making use of historical surveys that may not characterize the current malaria situation in the country. The produced maps and the estimates of the number of infected children illustrate an important synopsis of prevalence of malaria in the country. Therefore they can serve as a resourceful tool in planning interventions and a reference point in evaluating their impact in space and time.

Risk factor analysis was carried out using Bayesian variable selection within a geostatistical setting. This modelling approach identified not only the most important risk factors but also their functional form in order to build a parsimonious model with the best predictive ability. Bayesian variable selection has been implemented in malaria risk modelling by Giardina *et al.* [8] and Diboulo *et al*. [24]. Chammartin *et al*. [25] introduced Bayesian geostatistical variable selection for identifying functional forms of covariates in modelling neglected diseases however, to our knowledge rigorous modelling of covariate functional forms have not been used in the area of malaria mapping. The result indicated that in Nigeria, rainfall and NDVI are the most important drivers of malaria risk while temperature and altitude do not improve our ability to predict the risk.

The geographical distribution of the malaria risk estimates illustrated relatively high prevalence in every region of the country. The geostatistical model predicted higher disease risk (>40%) in some states both in southwest and in north central regions. Both regions have similar rainfall characteristics which create shallow water pockets, suitable breeding sites for *Anopheles gambiae*, the dominant mosquito vector in the country. Furthermore, in both regions there are many water bodies that are surrounded by vegetation, providing suitable habitat for *Anopheles funestus*, the second prevalent species in Nigeria. Malaria risk is relatively lower in the southern-most part of the country, which may be due to more rain in the region that could clear away breeding sites of the vector.

The distinct heaps of relatively high predicted parasitaemia risk around the survey locations might be explained by the weak spatial correlation in the observed prevalence which resulted in reduced smoothing of the predicted map. The predicted risk map was compared to a previous mapping effort across West Africa by Gemperli, *et al*. [5]. There were similarities in prevalence for most part of the southern Nigeria with the exception of Lagos where lower prevalence is obtained in this study which might be linked to more urbanization and ongoing interventions. Differences are present in the central north and northwest regions where higher and lower estimates were obtained respectively in this study. The malaria endemicity map produced by the malaria atlas project (MAP) [12] shows similar patterns to the present map especially in the north, apart from some areas in the north-east. In the south, MAP predicted higher risk in some parts of the southern-most regions principally around Cross river state. This might be connected to the inclusion of older children (2-10 years) in the MAP analysis. The estimates derived from this study when compared with the recently generated malaria endemicity map of Abdisalan *et al*. [13] shows resemblance in most part of the country aside the lake Chad area in the northeast and small fringes of Niger state in the central north where we predicted higher malaria risk.

The study findings indicated an increasing gradient of malaria risk with age, with the older children having the highest risk. The estimated negative association between socioeconomic status and malaria risk also confirms earlier reports [7-9]. The analysis showed that variation in the bed net coverage indicators across the country is not related to variation in the parasitaemia risk. However only data from one survey was available, therefore, changes in parasitaemia risk could not be estimated at a given region associated to intervention coverage levels.

A limitation of the survey is that it was carried out after the rainy season and, therefore, estimates may not reflect malaria risk during the highest transmission season. Rolling MIS [26] that adopt the standard cross-sectional evaluation tool into continuous monitoring can provide timely, accurate, sub-national, and district level burden estimates throughout the year. It was considered as a promising tool for monitoring short-term control progress in the course of its implementation in a district in Malawi, however its feasibility is unclear at national level.

## Conclusion

In conclusion, the predictive prevalence map depicts that malaria morbidity is still high in the entire country and variation in malaria intervention coverage indicators is not associated with variation in parasitaemia risk across the country. The coverage of key malaria interventions is still low and needs scaling up, which requires an increase of health expenditure by the federal government and an increase of awareness by the population on the benefit of bed net use.

## Additional files

Additional file 1:
**Geostatistical Model formulation.**
Additional file 2:
**Posterior median and 95% Bayesian Credible Intervals (BCI) of Model 1* and Model 2**.** *Model of malaria risk based on enviromental/climatic predictors. **Model of malaria risk inclusive of intervention after adjusting for climatic/environmental socioeconomic and demographic factors. Additional file 3:
**Estimates of the number of children under five years of age with parasitaemia at the state level.** Prev A: Population unadjusted prevalence. Prev B: Population adjusted prevalence.
