# Supplementary material for: Malaria risk in Nigeria: Bayesian geostatistical modelling of 2010 malaria indicator survey data
Source: Malar J. 2015 Apr 14;14:156. doi: 10.1186/s12936-015-0683-6 (PMC4404580; doi:10.1186/s12936-015-0683-6)
Supplement: Additional file 1: — Geostatistical Model formulation. [file 12936_2015_683_MOESM1_ESM.docx]

**Additional file1 : Geostatistical Model formulation**

Let *Y_ij_* indicate the malaria parasites status in child *i* at location *s_j_* (*j=1, …, n*).*Y_i j_* is assumed to follow a Bernoulli distribution, that is *Y_ij_* **~**Be ($p_{ij}$) , where $p_{ij}$ corresponds to parasitemia risk.

Also let $X_{ij}$= ($X_{ij}^{(1)}, X_{ij}^{(2)},\ldots X_{ij}^{(q)}$) ^T^ be the vector of predictors observed at location *s_j._*.We model the relationship between malaria risk and its potential predictors on the logit ($p_{\mathrm{ij}}$) = $X_{ij}\beta_{j}$ +$\varphi_{j}$, where $\beta_{j}$ are the regression coefficients. The spatial dependence was taken into account by location-dependent random effect $\boldsymbol{\varphi}$=$\left( \varphi_{1},\varphi_{2},\ldots\varphi_{n} \right)^{T}$which were considered to arise from a multivariate normal distribution with mean 0 and variance covariance matrix $Ʃ_{jk}$=$\sigma_{\varphi}^{2}\exp\left( -{\rho d}_{jk} \right)$, where $d_{jk}$ is the Euclidean distance between locations *s_j_* and *s_k_*, $\sigma_{\varphi}^{2}$ represent the spatial variance known as partial sill and $\rho$is a smoothing parameter that controls the rate of correlation decay with increasing distance. The range which defines the minimum distance at which spatial correlation between locations is below 5% is calculated by$3/\rho$ .We provide the estimate of the range parameter in km considering that 1 degree corresponds to 111.12km.

Bayesian variable selection^1–5^ was used to identify the most important predictors of parasitemia risk after taking into account the spatial correlation in the data at cluster level. This was done by introducing an indicator variable $\lambda_{j}$ suggesting the presence or absence of the corresponding $X_{j}$ covariate that is, $\beta_{j}$= $\lambda_{j}\alpha_{j}$ where $\alpha_{j}$ measure the effect size of $X_{j}$ . We assume a priori equal inclusion probabilities for all variables that is $\lambda_{j}\sim Be\left( \frac{1}{2} \right)$ and a mixture of normal distribution for$\alpha_{j}$ that is**,**  $\alpha_{j} \mathsf{I} \lambda_{j}\sim\left( 1-\lambda_{j} \right)N(0,\xi v^{2}$)+$\lambda_{j}N$(0,$v^{2}$). The parameter $v^{2}$ is a predetermined large variance (i.e. 1000) and $\xi$ is a small constant that shrinks $\beta_{j}$ towards zero when the covariate is not selected. We also introduce a separate indicator $I_{m}$ to select from linear or categorical form of the climatic factors. We assume $\beta_{jm1}$and $\beta_{jm2}$ represent the coefficients corresponding to the linear and categorical forms of the *j* predictor respectively, that is $\beta_{j}$=$I_{m}\beta_{jm1}$+ (1−$I_{m}$)$\beta_{jm2}$ with $I_{m}$ assuming a Bernoulli distribution. The selected model was fitted assuming a vague normal prior distribution for $\beta_{j}$,that is $\beta_{j}\sim$N (0,100).

Prior distributions were assigned to $\sigma_{\varphi}^{2}$ and *,*$\rho$ to complete the model specification. Inverse Gamma distribution was chosen for spatial correlation parameter $\sigma_{\varphi}^{2}$ that is, *p* ($\sigma_{\varphi}^{2}$) = Gamma(0.001,0.001). Uniform prior distribution assuming spatial correlation lower than 0.05 as negligible was chosen for 𝜌, that is *p* (𝜌 **) =** Uniform(-log(0.05)/d_max_,-log<(0.05)/d_min_) where d_max_ and d_min_ are the maximum and mininum (non-zero) Euclidean distance between the survey locations. To assess sensitivity of the estimates to the prior distributions of the spatial parameters, we re-fitted the models using a more informative prior distribution for $\sigma_{\varphi}^{2}$, that is *p* ($\sigma_{\varphi}^{2}$) **~** Gamma(2.01,1.01) and the following prior for 𝜌*, p* (𝜌 **) =** Uniform(-log(0.01)/d_max_,-log<(0.01)/d_min_).The model was fitted in WinBUG1.4 (Imperial College and Medical Research Council London, United Kingdom) using Markov Chain Monte Carlo (MCMC) simulation. Linear predictors were centered to obtain well-behaved correlation structure and reduce the computation time of MCMC algorithm^6^. The variable selection was carried out only for the environmental/climatic predictors to identify the most important predictors. Assessment of predictive performance of models were made by calculating the Mean Absolute Error (MAE) which provides information on model accuracy using the average of absolute distances between observed and predictive posterior distribution values, that is MAE$=\frac{1}{k}\sum_{i=1}^{k} \sum_{j=1}^{m} \left| \hat{p}_{ij}-p_{i} \right|$ where *k* is no of test locations and *m* is the size of sample drawn from the posterior predictive distribution at the test site. Also $\hat{p}_{ij}$ is the *j* sample of the predicted posterior distribution at the test site and $p_{i}$ is the observed prevalence at the test site.

1. Ishwaran, H. & Rao, J. S. Spike and slab variable selection: Frequentist and Bayesian strategies. *Ann. Stat.* **33,** 730–773 (2005).

2. Chammartin, F. *et al.* Statistical methodological issues in mapping historical schistosomiasis survey data. *Acta Trop.* **128,** 345–352 (2013).

3. Dellaportas, P., Forster, J. J. & Ntzoufras, I. On Bayesian model and variable selection using MCMC. *Stat. Comput.* **12,** 27–36 (2002).

4. Dellaportas, P., Forster, J. J. & Ntzoufras, I. Bayesian variable selection using the Gibbs sampler. *Biostat.-BASEL-* **5,** 273–286 (2000).

5. O’Hara, R. B. & Sillanpää, M. J. A review of Bayesian variable selection methods: what, how and which. *Bayesian Anal.* **4,** 85–117 (2009).

6. Banerjee, S., Gelfand, A. E., Knight, J. R. & Sirmans, C. F. Spatial Modeling of House Prices Using Normalized Distance-Weighted Sums of Stationary Processes. *J. Bus. Econ. Stat.* **22,** 206–213 (2004).
