# Supplementary material for: Malaria risk in Nigeria: Bayesian geostatistical modelling of 2010 malaria indicator survey data
Source: Malar J. 2015 Apr 14;14:156. doi: 10.1186/s12936-015-0683-6 (PMC4404580; doi:10.1186/s12936-015-0683-6)
Supplement: Additional file 2: — Posterior median and 95% Bayesian Credible Intervals (BCI) of Model 1* and Model 2**. *Model of malaria risk based on enviromental/climatic predictors. **Model of malaria risk inclusive of intervention after adjusting for climatic/environmental socioeconomic and demographic factors. [file 12936_2015_683_MOESM2_ESM.doc]

Additional File 2

|  | | ***Model 1** | | | ****Model 2** | | |  |
| --- | --- | --- | --- | --- | --- | --- | --- | --- |
|  |
| **Variables** | | **OR(95% BCI)** | | | **OR(95% BCI)** | | |  |
| **NDVI** | | 2.01 (1.56, 2.60) | | | 1.56 (1.21, 1.99) | | |  |
| **Rain** | | 0.57 (0.44, 0.75) | | | 0.72 (0.57, 0. 91) | | |  |
| **Area type** | |  | | |  | | |  |
| rural | |  | | | 1 | | |  |
| urban | |  | | | 0.43 (0.28, 0.65) | | |  |
| **Socioeconomic Index** | |  | | |  | | |  |
| Most poor | |  | | | 1 | | |  |
| Very poor | |  | | | 1.12 (0.86, 1.44) | | |  |
| Poor | |  | | | 1.19 (0.89, 1.59) | | |  |
| Less poor | |  | | | 1.00 (0.72, 1.39) | | |  |
| Least poor | |  | | | 0.51 (0.35, 0.75) | | |  |
| **Age** | |  | | |  | | |  |
| 0-1 | |  | | | 1 | | |  |
| 1-2 | |  | | | 1.35 (1.05, 1.76) | | |  |
| 2-3 | |  | | | 1.93 (1.50, 2.50) | | |  |
| 3-4 | |  | | | 2.34 (1.82, 3.02) | | |  |
| 4-5 | |  | | | 2.76 (2.15, 3.55) | | |  |
| **Proportion with**  **access to ITN in the household** | |  | | | 0.86(0.51,1.48) | | |  |
| **Proportion of children aged 0–59 months who slept under an ITN the night before the survey** | | |  | | | 0.91(0.57, 1.47) | |  |
| **Proportion of people who slept under an ITN in the night before the survey** | | |  | | | 0.92(0.50, 1.67) | |  |
| **Spatial Parameters** | **Posterior median**  **Model 1** | | | **95% BCI** | | | **Posterior median**  **Model 2** | **95% BCI** |
| **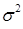**   |  | | --- | | 1.56 | | | (1.20, 2.04) | | | 1.34 | (1.02, 1.76) |
| Range(km) | 5.87 | | | (1.54, 60.00) | | | 2.14 | (1.12, 45.00) |
