# Supplementary material for: Malaria risk in Nigeria: Bayesian geostatistical modelling of 2010 malaria indicator survey data
Source: Malar J. 2015 Apr 14;14:156. doi: 10.1186/s12936-015-0683-6 (PMC4404580; doi:10.1186/s12936-015-0683-6)
Supplement: Additional file 3: — Estimates of the number of children under five years of age with parasitaemia at the state level. Prev A: Population unadjusted prevalence. Prev B: Population adjusted prevalence. [file 12936_2015_683_MOESM3_ESM.doc]

**Additional file 2**

| **Region** | **State** | **Prev A** | **Population of under 5 children** | **Estimated number of infected children** | **95% CI** | **Prev B** |
| --- | --- | --- | --- | --- | --- | --- |
| **North Central** | **Benue** | 29.73% | 784875 | 222340 | (200190, 244491) | 28.33% |
|  | **Kwara** | 42.37% | 449638 | 151621 | (131421, 71821) | 33.72% |
|  | **Kogi** | 41.50% | 561582 | 213304 | (188056, 38552) | 37.98% |
|  | **Nasarawa** | 37.56% | 340392 | 119592 | (102802, 36383) | 35.13% |
|  | **Niger** | 39.11% | 735787 | 256231 | (233893, 278568) | 34.82% |
|  | **Plateau** | 31.88% | 608271 | 150934 | (142149, 59720) | 24.81% |
|  | **FCT** | 34.31% | 233252 | 60270 | (53799, 66741) | 25.84% |
| **North East** | **Adamawa** | 28.51% | 566017 | 147375 | (135450, 59299) | 26.04% |
|  | **Bauchi** | 33.92% | 915634 | 281832 | (251864, 11799) | 30.78% |
|  | **Borno** | 33. 01% | 760560 | 329545 | (318480, 40610) | 43.33% |
|  | **Gombe** | 29.74% | 415270 | 108147 | (94212, 122082) | 26.04% |
|  | **Taraba** | 26.02% | 413337 | 95666 | (88832, 102500) | 23.14% |
|  | **Yobe** | 39.90% | 408299 | 143671 | (117597, 69744) | 31.54% |
| **North West** | **Jigawa** | 32.61% | 743497 | 245984 | (201408,290560) | 33.08% |
|  | **Kaduna** | 30.00% | 1087823 | 306971 | (274214, 39727) | 28.22% |
|  | **Kano** | 32.98% | 1661333 | 413643 | (359266, 468019) | 24.90% |
|  | **Katsina** | 31.31% | 1037799 | 312991 | (269742, 56240) | 30.16% |
|  | **Kebbi** | 33.17% | 547728 | 178198 | (155795, 200601) | 32.53% |
|  | **Sokoto** | 29.23% | 758304 | 241214 | (196928, 85499) | 31.81% |
|  | **Zamfara** | 30.24% | 662075 | 195519 | (167378, 223658) | 29.53% |
| **South East** | **Abia** | 34.34% | 552137 | 170719 | (131633, 209805) | 30.91% |
|  | **Anambra** | 18.95% | 753168 | 116883 | (99151, 134615) | 15.52% |
|  | **Ebonyi** | 25.50% | 440488 | 117079 | (81696, 152462) | 26.58% |
|  | **Enugu** | 28.30% | 638279 | 144983 | (120597,169370) | 22.71% |
|  | **Imo** | 24.23% | 777127 | 149944 | (116532, 83355) | 19.29% |
| **South South** | **Akwa Ibom** | 24.71% | 777083 | 183459 | (148354, 218564) | 23.61 |
|  | **Bayelsa** | 28.11% | 293027 | 73662 | (61359, 85965) | 25.14% |
|  | **Cross River** | 21.84% | 508781 | 113384 | (94271, 132496) | 22.29% |
|  | **Delta** | 25.40% | 838073 | 178389 | (153985, 202794) | 21.29% |
|  | **Edo** | 41.85% | 609027 | 216539 | (190803, 42276) | 35.55% |
|  | **Rivers** | 22.39% | 835356 | 160600 | (237741, 261026) | 19.23% |
| **South West** | **Ekiti** | 41.24% | 522684 | 175495 | (140018, 10972) | 33.58% |
|  | **Lagos** | 19.63% | 1765136 | 114114 | (94296, 133933) | 6.46% |
|  | **Ogun** | 33.50% | 778465 | 180659 | (153966,207352) | 23.21% |
|  | **Ondo** | 40.98% | 553129 | 211170 | (181103, 241236) | 38.18% |
|  | **Osun** | 47.74% | 655875 | 276857 | (222237, 331476) | 42.21% |
|  | **Oyo** | 38.78% | 977540 | 345095 | (291686, 398504) | 35.30% |
